# An update on ox-LDL-inducing vascular smooth muscle cell-derived foam cells in atherosclerosis

**DOI:** 10.3389/fcell.2024.1481505

**Published:** 2024-10-25

**Authors:** Jingjing Guo, Laijing Du

**Affiliations:** ^1^ Luoyang Key Laboratory of Cardiovascular Science, The First Affiliated Hospital, and College of Clinical Medicine of Henan University of Science and Technology, Luoyang, China; ^2^ Department of Cardiology, Henan Key Laboratory of Cardiovascular Science, The First Affiliated Hospital, and College of Clinical Medicine of Henan University of Science and Technology, Luoyang, China

**Keywords:** VSMCs, foam cell, atherosclerosis, ox-LDL, cholesterol metabolism

## Abstract

Excess cholesterol accumulation induces the accumulation of foam cells, eventually accelerating atherosclerosis progress. Historically, the mechanisms of macrophage-derived foam cells have attracted attention because of their central role in plaque development, which was challenged by lineage tracing in union with single-cell sequencing (sc-seq). Accumulated studies have uncovered how vascular smooth muscle cells (VSMCs) proliferate and migrate to the vascular intima and accumulate, then transform into foam cells induced by surplus lipids, finally accounting for 30% to 70% of the total foam cells within the plaque of both mice and humans. Therefore, the mechanisms of VSMC-derived foam cells have received increasing attention. The review intends to summarize the transformation mechanism of VSMCs into foam cells induced by oxidized low-density lipoproteins (ox-LDL) in atherosclerosis.

## Introduction

Atherosclerosis is a widespread and critical public health issue that is one of the leading causes of death globally (Barquera et al., 2015). In the presence of oxidized low-density lipoproteins (ox-LDLs), subendothelial vascular smooth muscle cells (VSMCs) in the human aorta increase the expression of macrophage marker cluster of differentiation 68 (CD68) and decrease SMC marker α-smooth muscle actin (α-SMA) expression (Andreeva et al., 1997). With the help of multicolor-labeling and random recombinant fluorescent transgene mice technology, accumulated studies found that the plaque comprised macrophage-like cells (CD68, galectin-3 (LGALS3/MAC2), and lysosomal associated membrane protein 2 (LAMP2/MAC3)), which came from a small subpopulation of VSMCs (Chappell et al., 2016; Jacobsen et al., 2017; Misra et al., 2018). Recently, studies found that a minimum of 50% to 70% of foam cells were individually redifferentiated from VSMCs in human coronary artery and apolipoprotein E-deficient mice plaques (Wang Y. et al., 2019; Allahverdian et al., 2014). Therefore, the importance of foam cells that are derived from VSMCs, especially in the progression of lesions, has been prominently highlighted (Pan et al., 2023).

**FIGURE 1:**
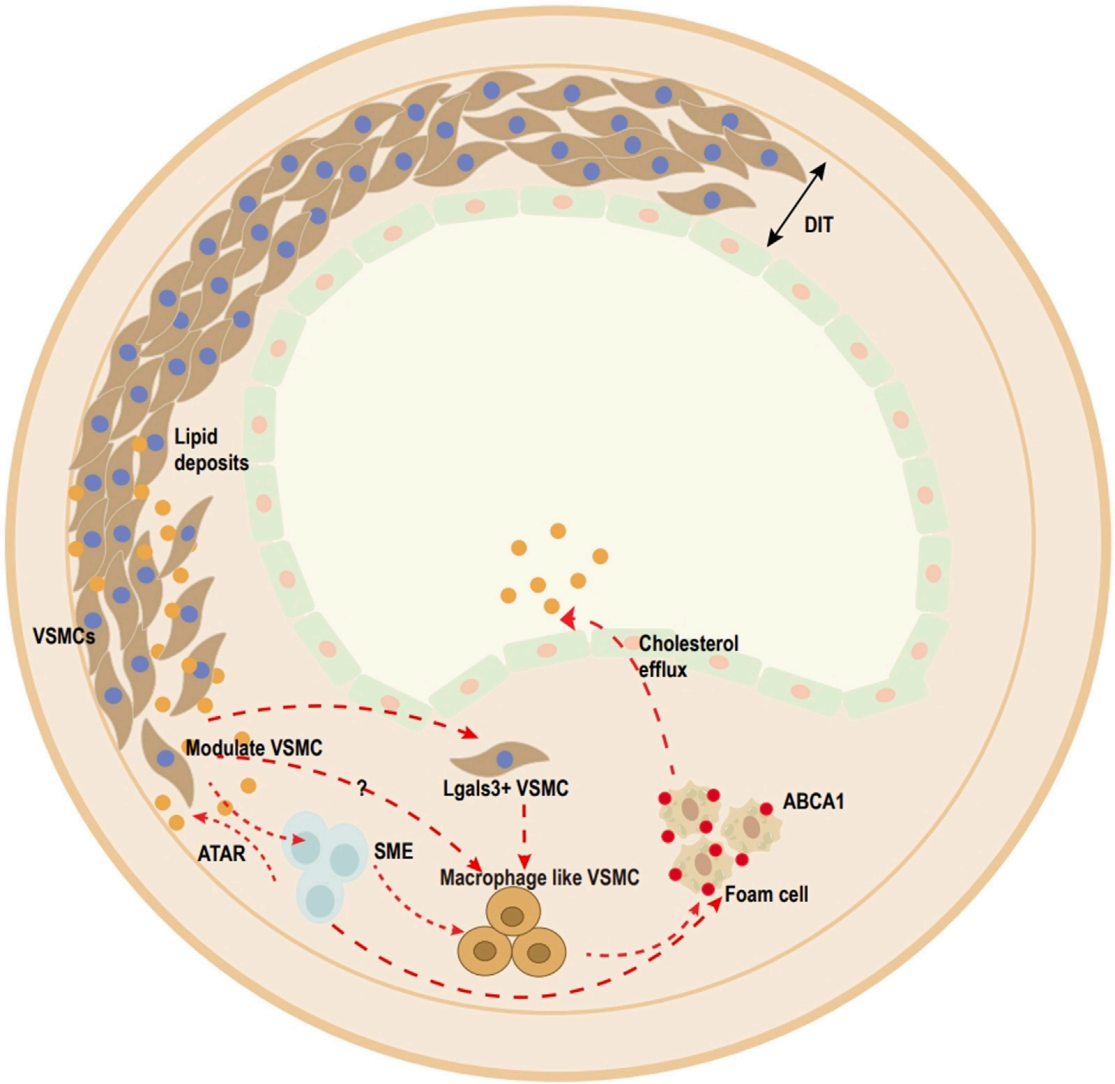
Overview of vascular smooth muscle cell (VSMC)-derived foam cell transition within media and atherosclerotic lesions (human). Ox-LDL uptake by VSMCs. Once accumulated in excess, contractile VSMCs transform into transitional pluripotent cells, including lgals3^+^VSMCs, SMC-derived intermediate cells (SEM), and macrophage-like SMCs. Pre-existing SMCs can be divided into (SMA)^+^ cells and predominantly (SMA)^−^ cells, and most plaque cells come from the clonal expansion of a single pre-existing (SMA)^+^ cell. SEM cells, which are SMC-derived intermediate cells, are multipotent and can differentiate into macrophage-like cells and return toward the SMC phenotype after treatment with all-trans retinoic acid (ATRA). Lgals3^+^-VSMCs also acquire a macrophage-like phenotype to become foam cells.

VSMCs are an integral part of the vascular media and exhibit high phenotypic plasticity (Alencar et al., 2020; Grootaert and Bennett, 2021). Much ox-LDL migrates into the subendothelial space. Then, ox-LDL is transferred into VSMCs through lectin-like oxidized low-density lipoprotein 1 (LOX-1), type A scavenger receptor (SR-A), platelet glycoprotein 4 (CD36), CXCL16/SR-PSOX, and other scavenger receptors, and redifferentiates into foam cells. During this process, VSMCs decrease the expression of VSMC markers (myosin heavy chain 11 (MYH11), α-SMA, and calponin 1) and increase the expression of macrophage markers (CD68, galectin, and Mac-2) (Wang Y. et al., 2019; Allahverdian et al., 2014; Rong et al., 2003; Wågsäter et al., 2004) (Figure 1). However, the process of how VSMCs transform into foam cells remains unclear. Therefore, the mechanism of VSMC-derived foam cell formation is discussed in this review, with an emphasis on recent developments.

### Origin of VSMC-derived foam cells

VSMCs are the core members of the medial arteries. Previously, due to the failure to trace VSMCs during the phenotypic switch, the important role of VSMC-derived foam cells was ignored (Bennett et al., 2016). Utilizing tamoxifen-inducible CreERT2 recombinase driven by VSMC-specific promoters (Tagln or Myh11), studies have revealed that VSMC-derived foam cells in advanced lesions upregulate the expression of macrophage markers and downregulate the expression of VSMC markers (Feil et al., 2014). Immunostaining for detecting VSMCs based on VSMC markers leads to ignoring more than 80% of VSMC-derived cells in plaque in a mouse model (Shankman et al., 2015). Dubland and Francis found approximately 50% of foam cells came from the redifferentiation of human VSMCs (Shankman et al., 2015). Ashish Misra et al. proposed that pre-existing SMCs divide into (SMA)^+^ cells and predominantly (SMA)^−^ cells and that most plaque cells originate from the proliferation of a single (SMA)^+^ cell. However, in the aortic media, the progenitor cells or cell pools marked with VSMC markers that participated in the formation of early atherosclerotic plaques have not yet been identified (Misra et al., 2018).

With the help of sc-seq technologies, the phenotypic heterogeneity of foam cells derived from VSMCs has been revealed (Pan et al., 2020). For example, SEM (VSMC-derived intermediate cells), which are intermediate cells derived from VSMCs, exhibit multipotency, differentiating into fibrochondrocyte-like and macrophage-like cells, while likewise reverting to the VSMC phenotype (Pan et al., 2020). Recently, Austin et al. analyzed VSMC transcriptomic single-cell data, especially those originating from models of atherosclerotic mice. The result indicated that Mac-2 is a transitioning phenotype during reprogramming from VSMCs to macrophage-like cells. However, intermediate states and the path of the phenotype switch of VSMCs, re-differentiating into macrophage-like cells, are still inadequate (Conklin et al., 2021).

It is still unclear whether VSMCs must first exhibit characteristics of macrophages during the process of transforming into foam cells or whether they begin to display these characteristics only after the transformation. Huize Pan and colleagues also reported that macrophage-like cells originating from VSMCs emerged later in lesions than resident or myeloid-derived macrophages. This finding, based on single-cell genomic analysis and SMC lineage tracing, may be questioned because of the diversity of the mice model as well as variances in the preparation techniques of single cells (Pan et al., 2020).

The detailed landscape of the VSMC-derived foam cells induced by ox-LDL in plaque needs further study.

### Consequences of VSMC-derived foam cell

The fate of foam cells, including apoptosis, autophagy, necrosis, and pyroptosis, plays an important role in regulating the formation and stability of atherosclerotic lesions. VSMC-derived foam cells mainly comprise the neointima of plaque (Bashore et al., 2024), and the death of VSMC-derived foam cells plays an important role in plaque stability. Apoptosis of VSMCs promotes the formation and expansion of necrotic cores and macrophage infiltration. Ox-LDL promoted apoptosis of VSMCs through activation of caspase cascade reaction, including Forkhead Transcription Factor O Subfamily Member 3(FOXO3)/ Apoptotic Protease Activating Factor 1(APAF1) and tensin homolog deleted on chromosome ten/ Phosphoinositide 3 kinase (PI3K)/Protein Kinase B(AKT), which led to plaques that are more prone to rupture (Yu et al., 2018; Huang and Kontos, 2002). In AS, efferocytosis, being eaten by the surrounding macrophages, is impaired in foam cells. Apoptosis of VSMCs is cleared by efferocytosis. The studies emphasized the clearance of apoptotic cells is crucial for the treatment of atherosclerosis (Yurdagul et al., 2020). Zheng Yin et al. suggested that EDIL3/Del-1 participated in the efferocytosis of apoptotic VSMCs in aortic dissection (Yin et al., 2024). Knockout of cyclin-dependent kinase inhibitor 2B impaired efferocytosis (Kojima et al., 2019). Recent studies found that VSMCs induced by thrombin acquired the ability to escape efferocytosis through upregulation of CD47 (Moldogazieva et al., 2019; Sun et al., 2022). Inefficient clearance of apoptotic VSMCs accelerated the formation of necrosis (Clarke et al., 2006). If apoptotic VSMCs fail to clear, necrosis, a non-programmed death form, will occur in VSMCs from atherosclerotic plaques (Bennett et al., 2016). However, ox-LDL alone fails to induce necroptosis of VSMCs (Dubland and Francis, 2016). Therefore, the strategy to enhance the clearance of VSMC-derived foam cells is important for the treatment of AS.

The deficiency of autophagy of VSMC-derived foam cells will be debated below. Adequate autophagy is crucial in the treatment of AS. Lipophagy, the targeted breakdown of lipid droplets, is a selective form of autophagy (Robichaud et al., 2021). It aims to degrade LDs and inhibits the formation of VSMC-derived lysosomes (Bai et al., 2022; Jeong et al., 2021; Yoo et al., 2021). Accumulated studies suggested that stearoyl-CoA desaturase-1 (SCD1) promoted the activation and nucleus translocation of transcription factor EB (TFEB), preventing the accumulation of LDs in VSMC-derived foam cells. This could potentially present a new direction for therapeutic interventions targeting atherosclerosis (Pi et al., 2019). IPARP1 was found to disrupt the molecular interaction between ATP-binding cassette G1 (ABCG1) and PLIN1, a critical association in cellular lipid metabolism, and enhanced that of P62 within LDs, which led to the accumulation of LDs (Lin et al., 2024). In addition, Ox-LDL induces the pyroptosis and ferroptosis of VSMCs, which promotes the formation of foam cells and plaque rupture (Wang et al., 2020; He et al., 2023; Zhou et al., 2021). However, the fates of VSMC-derived cells remain uncharacterized during plaque regression. The main reason is that it is difficult to trace the precise dynamic process and mimic a 3D environment *in vitro*.

### Regulation of cholesterol metabolism in VSMC-derived foam cells

**FIGURE 2:**
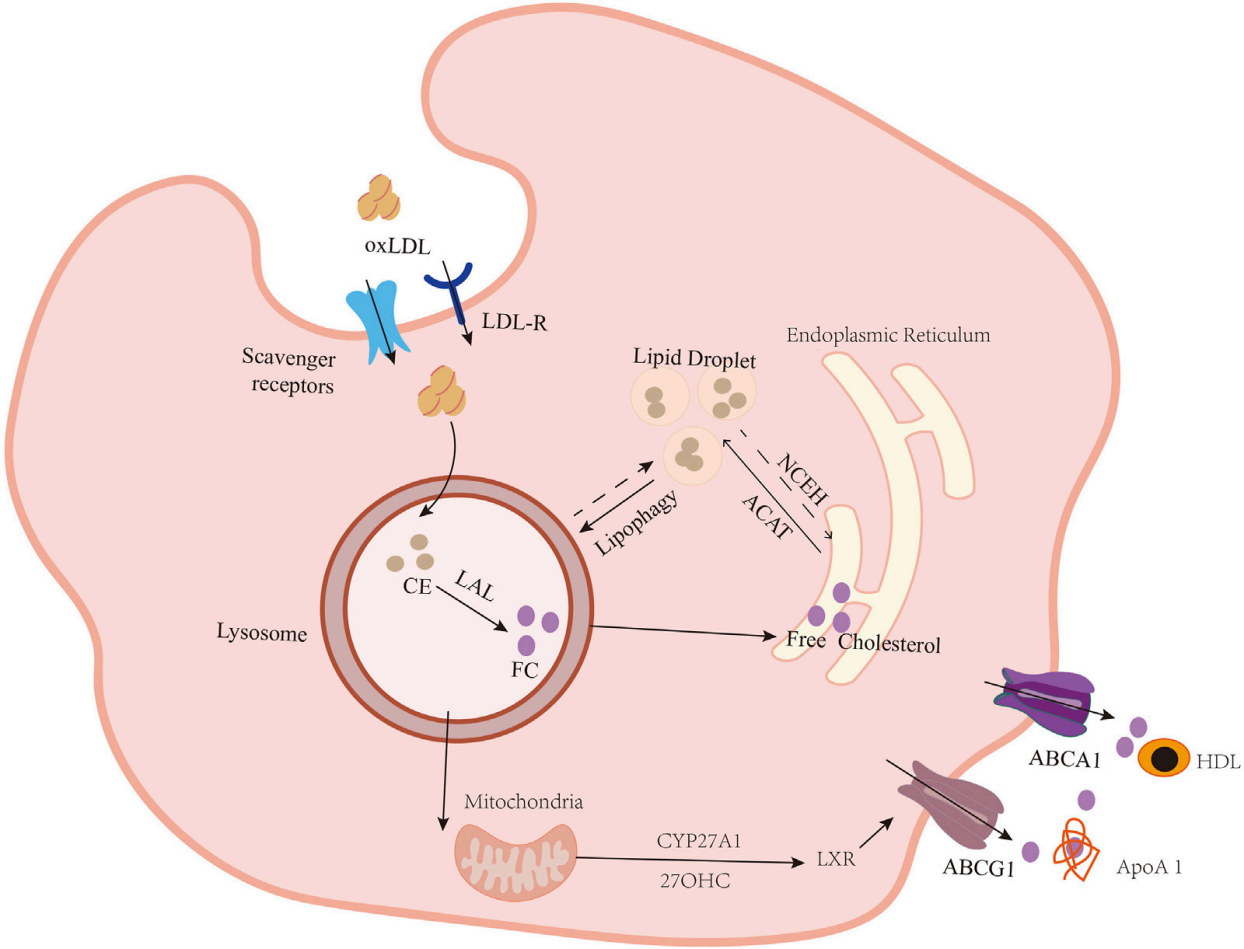
Formation of lipid-loaded VSMCs. VSMCs take up ox-LDL through lectin-like oxidized low-density lipoprotein 1 (LOX-1), type A scavenger receptor (SR-A), type B scavenger receptor (CD36), and CXCL16/SR-PSOX. Then, cholesteryl esters (CEs), the main form of cholesterol in lipoproteins, are hydrolyzed to free cholesterol (FC) at an acidic pH by lysosomal acid lipase (LAL), encoded by the LIPA (lipase A) gene in the lysosome. Once there is an FC surplus, they are shifted to the endoplasmic reticulum (ER). FC re-esterifies to CEs by acyl-coenzyme A: cholesterol acyltransferase-1 (ACAT1) and accumulates in the ER. Under normal conditions, CEs were stored in lipid droplets. If they accumulate in excess, VSMCs become foam cells. NCEH breaks down CEs and releases FC. FC is transported outside the cell via the ATP-binding cassette transporters (ABC) ABCA1 and ABCG1. ApoA-1 acts as a cholesterol transport receptor for ABCA1. Meanwhile, HDL serves as a recipient, collecting cholesterol transferred by ABCG1 and SR-BI. Lysosomally derived FC also provides the substrate for conversion to the oxysterol 27-hydroxycholesterol (27-OHC) in mitochondria by sterol-27-hydroxylase (CYP27A1). 27-OHC then travels to the nucleus, where it binds to liver X receptors (LXRs) on the promoter region of ABCA1 and other cholesterol export genes, including ABCG1, to upregulate their expression and facilitate efflux of the excess FC from cells.

Most cholesterol within lipoproteins exists as cholesteryl fatty-acyl esters (CEs). The equilibrium between CEs and free cholesterol (FC) is crucial for managing cholesterol levels in VSMCs (Liu et al., 2021). Ox-LDLs are uptaken via scavenger receptors and are hydrolyzed to FC by lysosomal acid lipase (LAL) in the lysosome (Kzhyshkowska et al., 2012; Park, 2014; Moore and Freeman, 2006). Excess FC is often repackaged into CEs by ACAT1 in the endoplasmic reticulum (ER) and then stored in cytoplasmic lipid droplets or effluxed through ATP-binding cassette (ABC) transporters (Mulas et al., 2014; Yin et al., 2014; Dubland and Francis, 2015; Chen L. et al., 2022) (Figure 2). In AS, the surplus of CEs leads to much FC retention in lysosomes. Once the processing capacity of lysosomes is surpassed, excess FC crystallizes. Cholesterol crystals cause overactive scavenger receptors (SR), and endocytosis forms numerous cytoplasmic lipid droplets and further promotes the formation of foam cells. In addition, cholesterol crystals in VSMC foam cells trigger apoptosis and expel cellular debris into the extracellular environment (Ho-Tin-Noé et al., 2017). Understanding cholesterol metabolism, therefore, is vital for AS treatment. This review mainly summarizes the role of ox-LDL in VSMC-derived foam cell formation from three aspects: the uptake of ox-LDL, the cyclic metabolism of cholesterol, and defective VSMC-derived foam cells, and also addresses the related regulatory mechanisms.

### Scavenger receptors

VSMCs take up modified LDLs, such as ox-LDL and aggregation-LDL (ag-LDL), via class B scavenger receptors (SR-B1 and SR-B2, or CD36) (Ben et al., 2015), SR-A (Shen et al., 2018), lectin-like oxidized low-density lipoprotein 1 (LOX-1 or SR-E1) (Sawamura et al., 1997) and the SR for phosphatidylserine and oxidized lipoprotein (SR-PSOX/CXCL16) (Moore and Freeman, 2006). Shu H et al. demonstrated that 75%–90% of modified lipoproteins are uptaken through CD36 and SR-A (Shu et al., 2022). In contrast, Manning-Tobin and colleagues uncovered that knockout of CD36 and SR-A among ApoE^−/−^ mice did not affect lesion size and macrophage/foam cell content (Deaton et al., 2009; Manning-Tobin et al., 2009).

#### SR-A

SR-A, belonging to the SR family, is expressed in VSMCs (Kodama et al., 1990; Daugherty et al., 1997; Li et al., 1995). It mainly interacts with ox-LDL and ac-LDL and changes with lipid internalization (Moore and Freeman, 2006). Suzuki et al. reported that knockout of SR-A1 in ApoE^−/−^ mice reduced cholesterol uptake and protected against atherosclerotic development (Suzuki et al., 1997). When cells are exposed to transforming growth factor-β 1 (TGF-β1) and ox-LDL *in vitro*, SR-AII and SR-AI levels are elevated in VSMCs (Li et al., 1995; Gong and Pitas, 1995; Yan et al., 2011). Krüppel-like factor 4 (KLF4) suppresses SR-A transcriptionally, and formononetin substantially attenuates the interplay between KLF4 and SR-A in VSMCs (Ma et al., 2020).

#### Scavenger receptor class B type I (SR-BI)

It is known that SR-BI is the core receptor for high-density lipoprotein (HDL) and mainly participates in cholesterol efflux (Phillips, 2014). Note that SR-BI also participates in the regulation of cholesterol influx. In endothelial cells (ECs), SR-BI is not only shown to inhibit the progress of AS by inducing the formation of nitric oxide but also promotes AS through enhancing LDL transcytosis (Mineo and Shaul, 2003; Yuhanna et al., 2001). However, whether SR-BI exhibits an atheroprotective effect in vascular tissue is unclear. Recent studies found SR-BI was a protective compensatory mechanism that maintained cholesterol homeostasis without ABCG1 (Oladosu et al., 2023).

#### CD36

CD36 was originally identified in the late 1980s and is in the SR family class B (Rao et al., 2014; Yu et al., 2013; Oquendo et al., 1989). Lesion development is significantly reduced in CD36^−/−^/ApoE^−/−^ mice (Febbraio et al., 2000). CD36 expression increases after treatment with ox-LDL, high glucose, or oleic acid, which enhances lipid accumulation in human coronary VSMCs. (Ma et al., 2011; Schlich et al., 2015; Xue et al., 2010). Accumulated studies found triggering receptor expressed in myeloid cells 2 (TREM2) expressions were raised in plaques (Depuydt et al., 2020; Cochain et al., 2018; Willemsen and de Winther, 2020; Guo et al., 2023). Ox-LDL activated PI3K/AKT, p38 mitogen-activated protein kinases (MAPK), and c-Jun N-terminal kinase (JNK) signaling pathways and enhanced the expression of HDGF and TREM2, which upregulated the expression of LOX-1 and CD36 (Guo et al., 2023). Meanwhile, ox-LDL induced Wnt Family member 5A (WNT5A) autocrine signaling and then activated disheveled segment polarity protein 2 (DVL2)/JNK signaling, which raised the level of CD36 (Ackers et al., 2020). Urszula Rykaczewska et al. found that Bcl2-associated transcription factor 1-ribosomal protein S8-tetraspanin-9-CD36 participated in the regulation and fate of VSMCs transforming into foam cells (Rykaczewska et al., 2022).

#### LOX-1

LOX-1, which accelerates foam cell production, is expressed in VSMCs (Sawamura et al., 1997) and accounted for 5%–10% of ox-LDL uptake (Mehta et al., 2007; Schaeffer et al., 2009). Ox-LDL induces VSMC transformation to foam cells through mtROS/c-Fos/LOX-1, which promotes ox-LDL uptake (Miao et al., 2022). In contrast, Zhang W et al. found ox-LDL suppressed the activity of non-receptor tyrosine kinase on the alpha1 C subunit (CACNA1C) and the L-Ca channel subunit, which led to downregulation of LOX-1 and subsequently promoted the proliferation, migration, and foam cell formation. Importantly, genistein mitigated these effects (Zhang et al., 2022). Zheng et al. also suggested that ox-LDL promoted autophagic degradation of LOX-1 (Zheng Y. et al., 2023). Deng et al. found that LKB1 participated in the regulation of the expression of LOX-1, which activated sirtuin 6 (SIRT6) through direct phosphorylation and then histone deacetylation of the promoter of LOX-1 (Deng et al., 2023). Myotoxin III, which is consistent in structure and function with phospholipase A2, upregulated the expression of SR-A and LOX-1 through PPAR-γ and PPAR-β/δ and enhanced intake of acetylated low-density lipoprotein (ac-LDL) in VSMCs (Giannotti et al., 2019). *Poria coco* polysaccharides inhibited the expression of LOX-1 induced by ox-LDL (Zhao et al., 2020). However, LOX-1 activation promoted oxidative stress, yielding higher ox-LDL production, thereby enhancing cycle self-amplification (Xu et al., 2013).

#### SR-PSOX/CXCL16

SR-PSOX/CXCL16, a class G member of the SR family, is expressed in VSMCs in human AS plaques and primarily binds and internalizes the ox-LDL (Zanoni et al., 2018) (Wågsäter et al., 2004; PrabhuDas et al., 2017; Minami et al., 2001; Hofnagel et al., 2002). IFN-γ notably stimulates CXCL16 expression in human VSMCs and increases ox-LDL uptake. However, this process does not influence CD36 and LOX-1 levels (Wågsäter et al., 2004).

#### Low-density lipoprotein receptor (LRP1)

Belonging to the LDLR family, LRP1 is expressed in VSMCs and is responsible for LDL uptake (Boucher et al., 2003; Llorente-Cortés et al., 2000). In VSMCs, knockout of LRP1 makes individuals more prone to cholesterol-induced atherosclerosis via the signaling pathways of platelet-derived growth factor receptor (PDGFR) and TGF-β (Boucher et al., 2003; Boucher et al., 2007). LRP1 has a protective function against atherosclerosis through modulating ATP-binding cassette A1 (ABCA1) expression via extracellular regulated protein kinases 1/2 (ERK1/2)/cytosolic phospholipase A2 (cPLA2/)arachidonic acid (Graves et al., 1996; Lin et al., 1992), an inhibitor of ABCA1 expression driven by liver X receptors (LXR) (DeBose-Boyd et al., 2001). Gordts PL et al. suggested LRP1 NPxYxxL motif is essential in LRP1’s anti-atherosclerotic function (Gordts et al., 2009). Therefore, atherosclerosis development can be mitigated via LRP1. In contrast, higher levels of LRP1 and intracellular lipid deposition were found in VSMCs that originated in advanced human plaques (Llorente-Cortés and Badimon, 2005). *In vivo*, ag-LDL induces expression of LRP1 in VSMCs and, in turn, promotes ag-LDL internalization through cluster II CR9 domain’s C-terminal half (Llorente-Cortés et al., 2002; Costales et al., 2015). Proprotein convertase subtilisin/kexin type 9 (PCSK9) regulates the metabolism of LDL through LDLR and facilitates foam cell formation via SNHG16/EZH2/H3K27me3/TRAF5 in VSMCs (Liu et al., 2023; Glerup et al., 2017). Thus, LRP1 may exert opposing effects in VSMCs. The dual function of LRP1 in the metabolism of LDL in VSMCs remains unclear.

#### Hydrolysis of CEs to FC in the lysosomes

Lysosomal acid lipase (LAL), encoded by lipase A gene (LIPA), is the sole enzyme for hydrolyzing CEs to FC in late endosomes/lysosomes, which is called lipophagy, or selective autophagy (Ouimet et al., 2011). In both human and mouse atherosclerosis, LAL prevents the overload of intracellular lipids and sustains cholesterol balance, which is the core progress to regulate VSMC-derived foam cell formation (Dubland and Francis, 2015; Dubland et al., 2021). However, Dubland and Jerome found impaired lysosomal function or deficiency of LIPA/LAL is an inherent characteristic that is not related to species in SMCs rather than a gene deficiency that is specific to a subset of individuals. Dubland et al. also suggested that the upregulation of LIPA or increasing activity of VSMC is an effective strategy for enhancing the processing of intracellular accumulated cholesterol in VSMC-derived foam cells. FOXO1 or TFEB is the activator of LIPA expression and the function of LAL. However, further studies are required to understand how upregulating these activators inhibits VSMC-derived foam cell formation.

#### Re-esterification of FC to CEs in the ER

ACAT1 catalyzes FC to CEs in the ER and is stored in cytosolic LDs (Suckling and Stange, 1985; Rudel et al., 2001). Accumulative evidence supports that many CEs accumulate in VSMCs or macrophage-derived foam cells and accelerate plaque formation (Ross, 1993). Higashimori et al. identified toll-like receptor 4 (TLR4) deficiency inhibited ACAT1 expression and formation of VSMC-derived foam cells induced by free cholesterol (Higashimori et al., 2011). Y-W Yin et al. further found that PPARγ inhibited the expression of ACAT1 through TLR4/MyD88/NF-κB (Yin et al., 2014). Bethany J Bogan et al. reported fatty acid synthase (FASN), which synthesized fatty acids, was upregulated in atherosclerotic human coronary arteries and induced foam cells derived from SMCs when exposed to cholesterol, which not only induced CD68 expression through KLF4/sterol O-acyltransferase 1 (SOAT1) but also inhibited ABCA1 expression to limit cholesterol efflux. FASN, therefore, acts in the biosynthesizing of fatty acids and functions in cholesterol esterification’s inverse association with efflux (Bogan et al., 2024).

#### Blocking LD formation by knocking down liquid droplet-associated proteins (LDAPs)

Liquid droplet-associated proteins (LDAPs), which are situated on the exterior of lipid droplets (LDs), are crucial for their assembly and breakdown processes (Yuan et al., 2012). PLIN2, belonging to the PAT family, is considered a scaffold protein for LD formation and is expressed in foam cells (Paul et al., 2008; Becker et al., 2010). During the process of lipid accumulation, PLIN2 relocates from the cell’s periphery to the cytoplasmic region, where it aids in the sequestration of long-chain fatty acids and the genesis of lipid droplets. (Gao et al., 2000). Myotoxin III (MT-III) facilitates the translocation of PLIN2 and PLIN3 from their usual repositories, underscoring the significance of these proteins in the assembly of lipid droplets within VSMCs (Giannotti et al., 2019). MT-III, an Asp49 sPLA2, induces LD formation in VSMCs. This biological effect is contingent upon the activity of key enzymes such as diacylglycerol O-acyltransferase (DGAT) and acyl-CoA: cholesterol acyltransferase (ACAT), as well as a contribution by peroxisome proliferator-activated receptors (PPARs), specifically the γ and β/δ subtypes.

#### Increasing CE hydrolysis in LDs

Neutral cholesteryl ester hydrolase (NCEH) hydrolyzes excess CEs to FC in LDs (Mulas et al., 2014; Yin et al., 2014; Dubland and Francis, 2015). This is the first step to start the reverse cholesterol transport (RCT). The FC from this process can be processed by two paths: one is re-esterification by ACAT1 in ER, and the other is efflux by ABC transporters (Sekiya et al., 2009; Escary et al., 1999). However, research related to the regulation of NCEH is limited in VSMCs. Additional research is necessary to determine the precise roles that NCEH plays in the level of FC as well as the process of cholesterol removal in VSMCs.

#### Cholesterol efflux through ABCG1 and ABCA1

During conditions of excessive lipid burden, ABCG1 and ABCA1 as transporters, along with SR-B, are responsible for a significant portion of cholesterol removal, accounting for approximately 60%–70% of the total efflux capacity (Jin et al., 2015) (Pownall et al., 2021). The cholesterol transporters ABCA1 and ABCG1 are recognized for their protective role against atherosclerosis (Phillips, 2014; Favari et al., 2015). ABCG1 is a mediator in the delivery of unbound cholesterol molecules to HDL particles, thereby participating in cholesterol transport mechanisms (Ouimet et al., 2019). An elevated expression of ABCG1 results in the increased transfer of unbound HDL particles, indicating its significant role in cholesterol efflux (Frambach et al., 2020). Nevertheless, mutation of ABCA1 in ApoE^−/−^ mice demonstrates minimal impact on the development of atherosclerotic plaques, suggesting a complex interplay of these factors in AS (Aiello et al., 2003). ABCA1 is vital because it promotes unesterified cholesterol transfer to ApoA-1 molecules that are deficient in lipids, thereby initiating the formation of HDL particles (Yvan-Charvet et al., 2007). Accumulated studies found that expression of ABCA1 was impaired in foam cells from VSMCs compared to foam cells that are derived from leukocytes (Wang Y. et al., 2019; Robichaud et al., 2022). The diminished capacity to expel surplus cholesterol is a principal factor driving VSMCs to become the primary source of foam cells in lesions (Lin et al., 2024).

It is known that KLF4 influences the phenotypic transition of intimal SMCs within atherosclerotic lesions (Kim et al., 2023). However, there has always been controversy regarding the relationship between KLF4 and ABCA1. Shankman and others suggested that the relationship between KLF4 and ABCA1 might be indirect or mediated by other factors (Shankman et al., 2015). In contrast, Bethany J Bogan et al. found that ABCA1 expression was a target of KLF4 (Bogan et al., 2024). Dubland et al. reported that oxysterol 27-hydroxycholesterol (27-OHC) is translocated into the nucleus, where it interacts with liver X receptors (LXRs) situated on the regulatory regions of the ABCA1 and ABCG1 genes. This binding event triggers an increase in the expression of these transporters, which, in turn, enhances the cellular mechanism to remove surplus free cholesterol (Dubland et al., 2021). Nuclear factor of activated T-cells 5 (NFAT5), which functions independently of calcineurin, is a key component of the NFAT family that induced lipid metabolism-related (Sod1, Plin2) and cholesterol efflux gene expression (ABCA1) (Kappert et al., 2021). Mao X et al. found substrate stiffness regulates cholesterol efflux through ABCA1 in VSMC-derived foam cells (Mao et al., 2021). *Ganoderma lucidum* spore powder (GLSP) has been shown to mitigate the progression of atherosclerosis and the associated calcification of the aorta by enhancing the cholesterol transport processes mediated by ABCA1/G1, which are crucial for maintaining cellular lipid balance (Zheng G. et al., 2023).

### Molecular mechanisms of cholesterol uptake, homeostasis, and efflux in VSMC-derived foam cells

#### Peroxisome proliferator-activated receptor gamma (PPARγ)

As a vital transcription factor, PPARγ regulates several genes involved in metabolizing lipids, glucose, and inflammation (Kamon et al., 2003; Tavares et al., 2007). As opposed to control wild-type (WT) mice, PPARγ/ApoE double knockout mice obtained more severe lesions associated with atherosclerosis, indicating PPARγ′s role in AS (Gao et al., 2020). Recent studies found that PPARγ inhibited inflammatory cytokines induced by LPS and ACAT1 expression via the TLR4/MyD88/NF-κB signaling pathway, which inhibited the VSMC-derived foam cell formation (Yin et al., 2014; Zhang et al., 2011). In addition, PARP1 facilitates the translocation of PLIN2 and PLIN3 and disrupts the interaction between ABCG1 and PLIN1, which leads to the accumulation of LDs (Lin et al., 2024). Myotoxin III regulates the relocation of lipid droplet metabolism-related proteins, including PLIN2 and PLIN3, and SR-A and LOX-1 expression through PPAR-γ in VSMCs (Giannotti et al., 2019). When VSMCs were changed into adipocytes, the expression of CCAAT enhancer-binding proteins (C/EBPα) and PPARγ increased (Davies et al., 2005; Porse et al., 2001). A recent study suggests C/EBPα acetylation induces PPARγ transactivation in macrophage-derived foam cells, suggesting that C/EBPα/PPARγ may participate in VSMC-derived foam cells, but this needs to be further confirmed (Gao et al., 2020).

#### TLR4

In cultured SMCs, TLR4 regulates the expression of inflammation induced by ox-LDL. Yin et al. discovered that activation of TLR4/MyD88/NF-κB by ox-LDL increased ACAT1 expression and promoted VSMC foam cell formation (Yin et al., 2014). Once TLR4 is knocked out, atherosclerotic plaque and foam cells derived from VSMCs induced by high lipids are inhibited. Xiao He et al. further found that MgCl_2_ inhibited the activation of the TLR4/NF-κB signaling pathway induced by ox-LDL, which attenuated pyroptosis of VSMC-derived foam cells (He et al., 2023). Following further study, Zhiyang Han et al. pointed out that HOXA1 may contribute to the positive feedback relationship between TLR4 and NF-κB (Han et al., 2023). Recently, Chen Z et al. showed that TLR4 regulated ox-LDL-induced VSMC foam cell production via the Sirt1/3 and Src pathways (Chen et al., 2021; Yang et al., 2015). TLR4 also participates in the regulation of ROS recruitment in VSMC-derived foam cells (Moldogazieva et al., 2019). Zhen Sun et al. suggested that macrophage CD36 can help TLR4 bind to foam cells (Sun et al., 2022). The above results suggest that TLR4 is involved in VSMC foam cell formation with ox-LDL inducement, which may be targeted in future research to develop effective therapeutic medications for AS.

#### P2RY12

A P2 receptor family member, P2RY12 includes P2RX/P2X ion channels and P2RY/P2Y G protein-coupled receptors (Walker et al., 2020). Recent studies showed levels of P2RY12 receptors in VSMCs were elevated during AS, as most VSMCs that are P2RY12-positive are situated proximate to foam cells and the plaque area (Pi et al., 2021). Conversely, in advanced AS, P2RY12 receptor activation mitigated the efflux of cholesterol as well as autophagy inhibition, which was through PI3K-AKT-mTOR *in vivo* and *in vitro* (Pi et al., 2021; Bao et al., 2024). Recent studies found that Gualou-Xiebai (GLXB), Geniposide, and Huanglian Jiedu, herbs used in Traditional Chinese medicine to treat AS, raised the levels of lipophagy via the P2RY12/PI3K/AKT signaling pathway (Lin et al., 2024; Pi et al., 2021; Bao et al., 2024).

#### mTOR signaling

Accumulated studies revealed the mechanistic target of rapamycin (mTOR), a downstream effector of PPARγ and P2RY12, participates in nutrient sensing and regulates autophagy in VSMC-derived foam cell formation (Kim et al., 2023; Pi et al., 2021; Zuo et al., 2023). Pi et al. suggested that the P2RY12 receptor inhibited autophagosomes through PI3K-AKT-mTOR in VSMCs, which were inhibited by inhibition of Cx43 (Pi et al., 2021; Qin et al., 2022). Kim et al. found PPARγ/mTORC2/FOXO3a-autophagy mitigated VSMC senescence (Kim et al., 2023). Wingless/Int-1 signaling further downregulates mTORC1/ribosomal protein S6 kinase 1 (p-P70S6) and limits VSMC cholesterol buildup in humans and atherosclerosis progression in mice (Awan et al., 2022). Bisdemethoxycurcumin (BDMC), possessing anti-oxidation, anti-inflammation, and other multi-pharmacological functions, promotes autophagy and mitigates lipid droplets via the PDK1/AKT/mTOR signaling pathway, which alleviates the accumulation of lipids in VSMCs. Therefore, it can be utilized in atherosclerosis treatment and avoidance as a therapeutic agent (Zuo et al., 2023).

#### TFEB

TFEB is a core transcription factor and regulates lysosome biogenesis and autophagy (Settembre et al., 2011). In VSMCs, activation of TFEB can modulate the migration and propagation of VSMCs, as well as reduce the formation of VSMC-derived foam cells (Pi et al., 2019; Wang YT. et al., 2019). Chen et al. found the sulfhydration of TFEB Cys212 increased nuclear translocation; LAPTM5 (biogenesis of lysosomes), LDLRAP1 (an autosomal recessive hypercholesterolemic gene known as LDL receptor cytoplasm tail binding protein), and Atg9A (associated with autophagosome formation) are its target genes (Chen Z. et al., 2022). TFEB also participates in the regulation of lysosomal acidification in macrophages. Recovery of lysosomal acidification is another challenge that VSMCs must face in lipid metabolism processes. Therefore, recovery of lysosomal acidification will be a strategy to rescue macrophage lysosomal dysfunction, and this strategy’s applicability to VSMC-derived foam cells remains to be investigated.

#### Epsins

Epsin1 and Epsin2 are members of the ubiquitin-binding endocytic adapter protein family. In macrophages, epsins are responsible for regulating EC reprograming to mesenchymal lipid uptake and cholesterol efflux in macrophages coming from AS plaque (Dong et al., 2023; Dong et al., 2020; Cui et al., 2023). Epsins are a positive regulatory factor in atherosclerotic lesion formation. Epsins promote the development of atherosclerosis by preventing the ubiquitination and degradation of LRP-1 in macrophages and regulating calcium release from the ER by promoting the proteasomal degradation of IP3R1 through its ubiquitin interaction motif (UIM) in endothelial cells (Dong et al., 2020; Brophy et al., 2019). Recent studies found that lack of Epsins induced VSMC transfer into myofibroblasts and ECs, which benefits the repair of vascular injury. This process was regulated through OCT4 and KLF4 (Wang et al., 2024). Nevertheless, whether epsins are involved in the disruption of lipid metabolism in VSMCs and VSMC-derived foam cell transformation and, if so, their regulatory mechanisms, has not been explored.

#### Others

**FIGURE 3:**
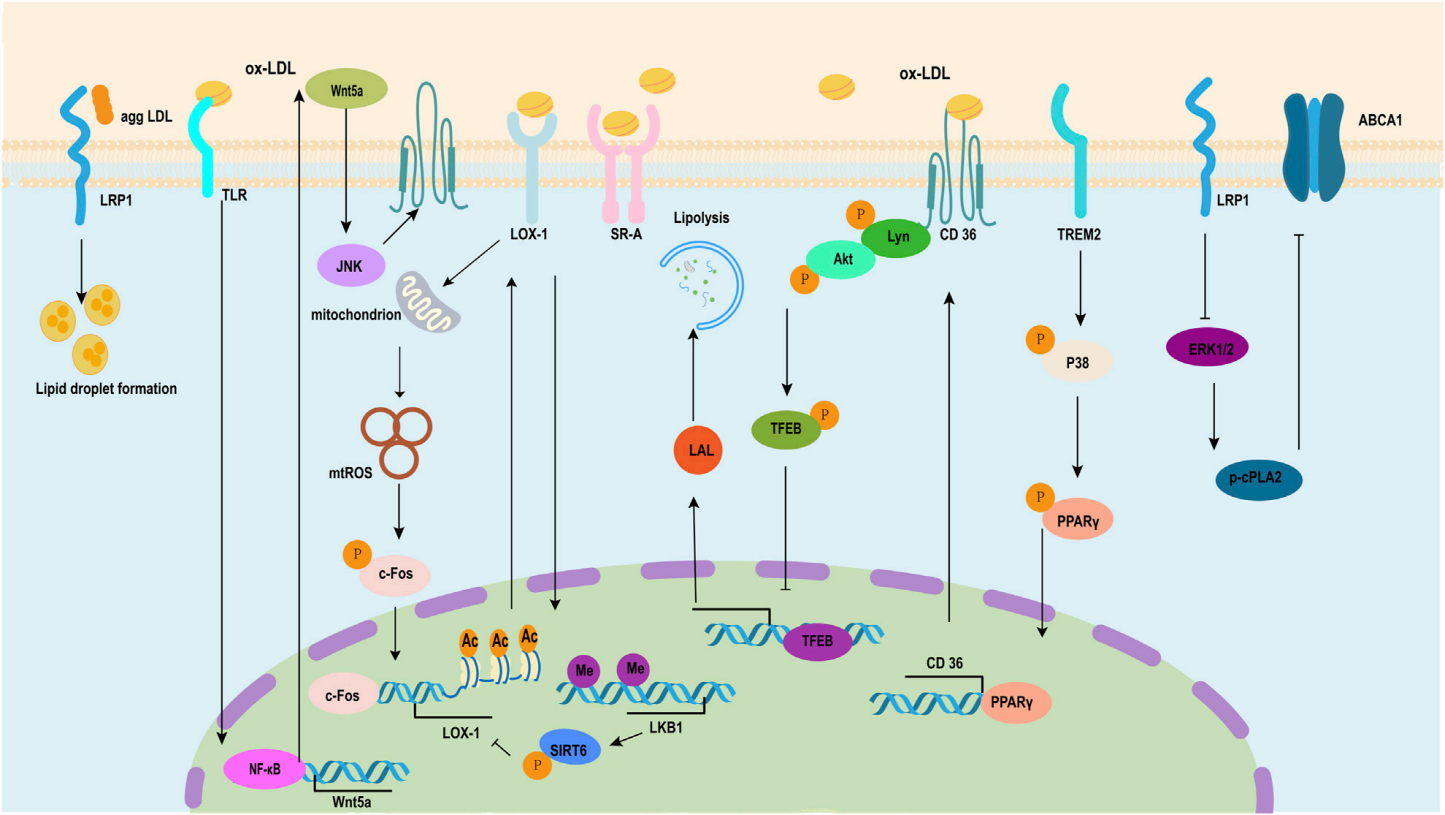
Pathway of the formation of VSMC-derived foam cells induced by ox-LDL. Low-density lipoprotein receptor-related protein (LRP) plays a dual role in the process of transforming smooth muscle cells into foam cells induced by ox-LDL, including inducing lipid droplet formation and inhibiting the expression of the ATP-binding cassette transporter (ABC) ABCA1 through ERK1/2/p-cPLA2. LRP1 plays a dual role in the process where high-fat stimulation transforms smooth muscle cells into foam cells. Ox-LDL induces WNT5A autocrine and then activates WNT5A/DVL2/JNK signaling, which enhances the expression of CD36. TREM2 upregulates the expression of the scavenger receptor CD36 in VSMCs by inducing the phosphorylation of p38 mitogen-activated protein kinase and PPARγ and increasing PPARγ nuclear transcriptional activity. Ox-LDL induces the direct phosphorylation of Lyn and AKT through CD36 and then downregulates the expression of LDL via TFEB. Ox-LDL induces VSMC transformation to foam cells through the mtROS/c-Fos/LOX-1 signaling pathway, which promotes ox-LDL uptake. LKB1 inhibits VSMC-derived foam cell formation and atherosclerosis via phosphorylation of SIRT6 and subsequent inhibition of LOX-1 expression. ERK1/2, extracellular signal-regulated kinases 1 and 2; cPLA2, cytosolic phospholipase A2; WNT5A, Wingless-Type mouse mammary tumor virus (MMTV) integration site family member 5A; DVL2, Disheveled segment polarity protein 2; JNK, c-Jun N-terminal kinase; TREM2, triggering receptor expressed in myeloid cells 2; PPARγ, peroxisome proliferator-activated-receptor gamma; AKT, protein kinase B; TFEB, transcription factor EB; LOX-1, Lectin-like oxidized low-density lipoprotein 1.

As a serine protease and a PCSK family member, proprotein convertase subtilisin/PCSK9 has an involvement in cardiovascular disease and dyslipidemia progression caused by atherosclerosis (Ragusa et al., 2021; Katsuki et al., 2022). A recent study revealed PCSK9 increased the lesions in mice fed with high-fat content and foam cell production in ox-LDL-treated VSMCs through lncRNA small nucleolar RNA host gene 16 (SNHG16)/histone-lysine N-methyltransferase enzyme (EZH2)/tumor necrosis factor receptor-associated factor 5 (TRAF5) (Liu et al., 2023). Homocysteine induces DNA hypermethylation in the promoter region of C1q/TNF-related protein 9 (CTRP9) via upregulated expression of DNA methyltransferase 1 (DNMT1) while also adversely regulating the deposition of lipids in VSMCs as mediated by ERs (Wang et al., 2023). Methyl-CpG binding protein 2 (MECP2)/ transcription factor homeobox A9 (HOXA9)/E3 ubiquitin ligase Peli1 participated in VSMC phenotypic switching induced by ox-LDL (Fu et al., 2023; Burger et al., 2022). A protein that binds to β-galactoside, galectin-1 (Gal-1) serves as an abdominal aortic aneurysm (AAA) and atherosclerosis therapeutic target. It enables more foam cells to be formed and increase mitochondrial dysfunction in VSMCs; such effects can be avoided via recombinant Gal-1 treatment (Roldán-Montero et al., 2022) (Figure 3).

## Conclusion

VSMCs are vital in atherosclerosis onset, progression, and all other stages. To date, it is known that VSMCs filled with lipids undertake expansion of clones and intima migration, from which lipid intake and foam cell phenotype acquisition occur and eventually become a part of the 30%–70% foam cells in atherosclerotic plaques in humans and mice. The origin of VSMC-derived foam cell formation is unclear. First, do VSMCs have predetermined mechanisms to regulate VSMC-derived foam cell migration to the intima from the medial layer? Second, what is the relationship between VSMC-derived foam cells and specific clones of VSMCs in the intima? Finally, do VSMC-derived foam cells continue to proliferate?

VSMCs have developed sophisticated mechanisms for cholesterol metabolism. The procession of VSMCs switching to foam cells is associated with disturbances of cholesterol metabolism-regulating CE and free cholesterol levels. First, ox-LDL induced upregulation of LOX-1 and CD36 and induced ox-LDL internalization. The levels of phagocytosis capacity and SR (CD36, LOX1) expression can undergo gene-level influence. They can be significant inter-individual susceptibility prognosticators in atherosclerosis and the formation of foam cells derived from VSMCs. Second, the imbalance of cholesterol esterification and free cholesterol was due to the upregulation of ACAT1 and downregulation of NCEH. Resounding proof exists that cholesterol trafficking is vital in intra-arterial-cell cholesterol accumulation in both early- and late-stage atherosclerosis. Lysosomes and vesicular movements emerged as central components in cholesterol trafficking. Such mechanisms in cellular biology and physiology in cholesterol sensing and lysosomal function are important targets in atherosclerosis treatment and avoidance. Foam cells originating from VSMCs have defective autophagy and expression of ABCA1 compared to macrophages in response to lipid loading, which contributes to cholesterol efflux impairment and dysfunctional lysosome accumulation. An in-depth study of the discrepancy between VSMC-derived foam cells and macrophage-derived foam cells will identify important targets for the treatment of atherosclerosis.
